# Analysis of multidrug-resistant Gram-negative pathogens (MRGN) in different areas of the healthcare system and their significance in the outpatient sector

**DOI:** 10.3205/id000105

**Published:** 2026-02-09

**Authors:** Cosima Berdin, Tobias Kaspers, Barbara Gärtner, Alexander Halfmann, Fabian K. Berger, Nina Walzer, Sören L. Becker, Sophie Schneitler

**Affiliations:** 1 Institute of Medical Microbiology and Hygiene, Saarland University Medical Center, Homburg, Germany; 2 National Reference Center for Clostridioides difficile, Homburg – Münster – Coesfeld, Germany; 3 Institute of Virology, Saarland University Medical Center, Homburg/Saar, Germany; 4 Institute for Medical Microbiology, Immunology and Hygiene, University Hospital Cologne, Germany

**Keywords:** drug resistance, COVID-19, ambulatory care, beta-lactam resistance, MDR

## Abstract

Given the global threat of increasing antibiotic resistance, risk factor detection of multi-resistant pathogens is particularly important. This is complicated by different definitions, using the international extended spectrum beta-lactamases (ESBL) definition and the German definition of multidrug-resistant Gram-negative pathogens (MRGN). Although the MRGN definition was primarily introduced for hospital hygiene measures, it is often used in outpatient or semi-inpatient areas. Due to the increasing numbers of outpatient treatments of the healthcare system, corresponding data is necessary for specific hygiene regulations.

This study provides MRGN and ESBL data based on a stool examination and a questionnaire evaluation in the period 07/2021–03/2022 of 231 outpatients of Saarland University Medical Center before traveling abroad.

There was a 3MRGN prevalence of 2.6% with five *Escherichia coli* and one *Klebsiella pneumoniae* and an ESBL prevalence of 5.6% with 13 ESBL *Escherichia coli*, four of which could also be classified as 3MRGN. These prevalences were compared with MRGN/ESBL prevalences in PubMed and Google Scholar in different areas of the German healthcare system in the period 2013–2024 at the federal state level. The selective literature search revealed geographical differences and missing prevalence data depending on the healthcare sector (outpatient/inpatient) and federal state.

Resistance data is often evaluated according to international standards, i.e. according to the ESBL definition. Outpatient MRGN prevalences are hardly known despite the increasing numbers of outpatients of the healthcare system. Due to the scarcity of outpatient data, our study from a travel medicine clinic provides interesting epidemiological data that should be considered in the context of the COVID-19 pandemic.

## Introduction

According to the World Health Organisation (WHO), the increase in antibiotic resistance is one of the ten greatest global threats to public health [1], with a WHO list from 2017 highlighting the risk posed by Gram-negative pathogens in particular [2]. The risk factors for acquiring resistance are multidimensional and the prevalence varies geographically [3]. The “One Health” concept highlights the connection between humans, animals and the environment, with data from human and veterinary medicine as well as environmental studies showing that the use of antibiotics leads to the selection of multi-resistant pathogens [4]. A 2015 report showed that outpatient prescriptions accounted for about 85% of overall antibiotic consumption in human medicine in Germany [5]. Regionally, a diverging risk factor can be assumed, as the use of antibiotics varies in different areas, including agriculture [6], [7].

In the USA, warnings were issued about increasing resistance during the COVID-19 pandemic [8], but Werner et al. reported a decrease in multidrug-resistant Gram-negative pathogens (MRGN) during the pandemic years in German intensive care units following rising colonisation rates until 2019 [4]. Further epidemiological data focusing on MRGN in other areas of the healthcare system, such as outpatient care, particularly with regard to undetected colonisation, are of great importance for the development of differentiated hygiene measures.

There are various classifications for Gram-negative pathogens. In German-speaking countries, the MRGN classification of the Commission for Hospital Hygiene and Infection Prevention (KRINKO) at the Robert Koch Institute (RKI) [9] is primarily used, whereas internationally, extended-spectrum beta-lactamases (ESBL) in particular are evaluated. 

The RKI defines ESBL as resistance to cefpodoxime and cefotaxime and/or ceftazidime, which can be inhibited by beta-lactamase inhibitors such as sulbactam or clavulanic acid (MIC_cephalosporin_/MIC_cephalosporin+inhibitor_≥8) [10]. Beta-lactamases can hydrolyse the beta-lactam ring of beta-lactam antibiotics and prevent binding to the transpeptidase (penicillin-binding protein) of the bacterial cell wall [11]. Depending on their hydrolysis capacity, there are different types such as penicillinases, cephalosporinases and carbapenemases [12]. 

The MRGN definition is based on in vitro resistance to key substances from four antibiotic groups (piperacillin (acylureidopenicillins), ceftazidime and/or cefotaxime (3^rd^/4^th^ generation cephalosporins), imipenem and/or meropenem (carbapenems) and ciprofloxacin (fluoroquinolones)) [9]. Resistance to three or four of the key substances is described as 3MRGN and 4MRGN [9]. Carbapenem resistance in 4MRGN can arise in addition to AmpC or ESBL formation and porin loss due to carbapenemases [9]. It is used in particular in hospitals for risk assessment, determining hygiene measures such as isolation or basic hygiene (e.g. surface disinfection) and establishing screening guidelines [9].

Inpatient data is primarily used to derive hygiene measures, which may be due, among other things, to the available data collection. Comparing data in inpatient and outpatient contexts is difficult due to different risk factors and patient groups. This is illustrated by studies on colonisation with Gram-negative pathogens during foreign travel [13], [14], [15].

The increasing shift towards outpatient care requires a better understanding of MRGN occurrence in the outpatient sector in order to develop risk-stratified hygiene measures. The aim of this study is to present outpatient MRGN data from one federal state and compare it with other published data from various areas of the German healthcare system. Based on this survey, the various outpatient regional MRGN resistance patterns will be analysed, highlighting the difficulties of a uniform survey using the example of classification patterns, and measures in the outpatient sector will be discussed. 

## Method

Resistance data were collected in the Travel and Tropical Medicine Outpatient Clinic at Saarland University Hospital from travellers who required a COVID-19 PCR test before travelling abroad between 1 July 2021 and 15 March 2022 using an MRGN risk questionnaire and a stool test before travel. 

The study was conducted with the approval of the responsible ethics committee (Saarland Medical Association, 205/21), in accordance with national law and the Declaration of Helsinki. All participants provided their consent for anonymous data collection.

The questionnaire consisted of 30 multiple-choice questions and three open-ended questions for general, personal information about future and past travel destinations and the duration of travel. To create the questionnaire, MRGN and ESBL risk factors in travellers from previous studies were evaluated and supplemented with additional questions focusing on MRGN (Table 1 (Tab. 1)). The questionnaire consisted of basic demographic questions (including age) as well as questions about medical history and travel behaviour. 

The stool sample was examined using chromogenic ESBL agar (CHROMagar ESBL/VRE ready plates; Mast Diagnostica). Growth was distinguished by different staining patterns and the morphology of the pathogens to identify different species. Relevant colonies were identified using matrix-assisted laser desorption/ionisation time-of-flight (MALDI-TOF) mass spectrometry (Bruker, Bremen). Sensitivity testing was performed using the MicroScan autoSCAN-4 system (Beckman Coulter) with a Neg multidrug-resistant MIC 1 (NMDR1) panel (Beckman Coulter). Evaluation was performed according to EUCAST 11.0 (European Committee on Antimicrobial Susceptibility Testing) criteria. In cases of technical measurement uncertainty, verification was performed using an epsilometer test (MIC Test Strips, Liofilchem^®^). An ESBL confirmation test was performed using the disc diffusion method “AmpC and ESBL Detection Set” (MASTDISCS^®^ Combi) if ESBL could not be reliably confirmed in the MicroScan autoSCAN-4 system.

The statistical analysis of group differences was performed using the SPSS programme (version 29.0 (IBM)) by means of logistic regression analysis, whereby odds ratios (OR), 95% confidence intervals (CI) and p-values were calculated. P-values were evaluated with a significance level of a=0.5% and tested bilaterally. If the cell counts were too small, the p-values of categorical data were determined using Fisher’s exact test. MRGN/ESBL colonisation was classified as a dependent variable in the regression analysis and the risk factors to be investigated were classified as coefficients (0=no, 1=yes). The 95% CI of prevalence was calculated using Stata Version 16.1 (Stata Corp.) with Agresti-Coull confidence intervals [16].

**Figure 1:**
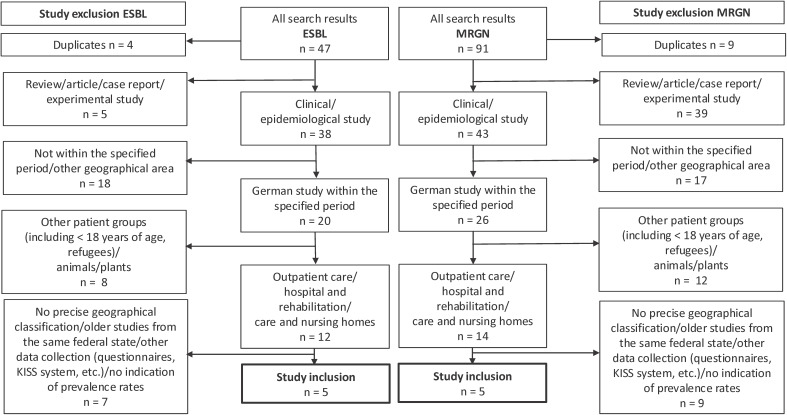
Flow chart of study inclusion ESBL/MRGN in the period 2013–2024 PubMed with filter “Humans”: search parameters “ESBL Germany Travel”, as of 25 June 2024 (19 results), search parameters “MRGN”, as of 7 July 2024 (45 results) Google Scholar: search parameter “ESBL Germany” in title, as of 10 July 2024 (28 results), search parameter “MRGN” in title, as of 10 July 2024 (46 results) Citations and patents were excluded.

**Figure 2:**
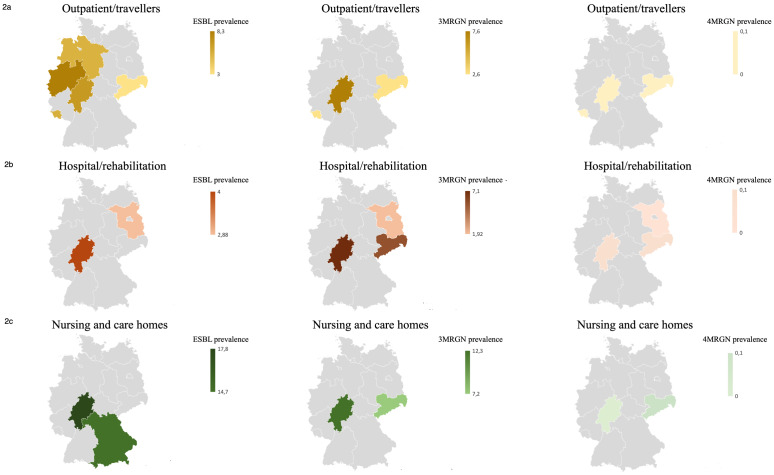
Consolidated figure of the literature study data on prevalences (%) of outpatient/travellers (2a), hospital/rehabilitation (2b) and nursing and care homes (2c) with illustrations of the resistance definitions ESBL (column 1), 3MRGN (column 2) and 4MRGN (column 3). The corresponding colour intensity reflects the respective evidence; if data is missing or there is no evidence of resistance for this area, grey is used as the background colour. The following studies, which were mapped at the federal state level, were taken into account for the creation of the graphs for the data collection period 2013–2024 (authors’ details including numerical reference, data can be requested from the authors): Heudorf et al. [26]; Hogardt et al. [20]; Kiefer et al. [45]; Meurs et al. [26]; Neumann et al. [22]; Schaumburg et al. [27]; Sommer et al. [24]; Steul et al. [21]; Symanzik et al. [25]; Valenza et al. [46]

The comparative analysis was performed using selective literature searches in PubMed and Google Scholar (Figure 1 (Fig. 1) and Figure 2 (Fig. 2)). The search parameters “ESBL Germany Travel” and “MRGN” and the filter “Humans” were used in PubMed and supplemented by a title search with the keywords “MRGN” or “ESBL Germany” in Google Scholar. Studies were searched for between 2013 and 2024 that examined geographically clearly assignable data on German ESBL/MRGN prevalences and could be assigned to the study settings outpatient/travellers, hospital/rehabilitation or nursing and care homes. Only studies that examined stool samples and/or (peri-)anal/rectal swabs from adult study participants were included. Studies were excluded if no specific geographical assignment to a federal state could be made. This did not apply to studies from the Rhine-Main area, which were assigned to the federal state of Hesse. If study data from the same area and federal state were available, the most recent study data were used. A total of twenty studies were excluded due to their focus on individual patient groups, such as refugees and pregnant women [17], [18] or agricultural workers [19]. The diagrams were created in Excel using @GeoNames, Microsoft, Tomtom (supported by Bing). 

## Results

The complete data set included 231 participants (380/60.79%) who planned trips lasting from one day to 280 days, predominantly female (n=126/54.5%) and with a median age of 39 [25^th^ to 75^th^ percentile: 27–56] (Table 1 (Tab. 1)).

**Figure 3:**
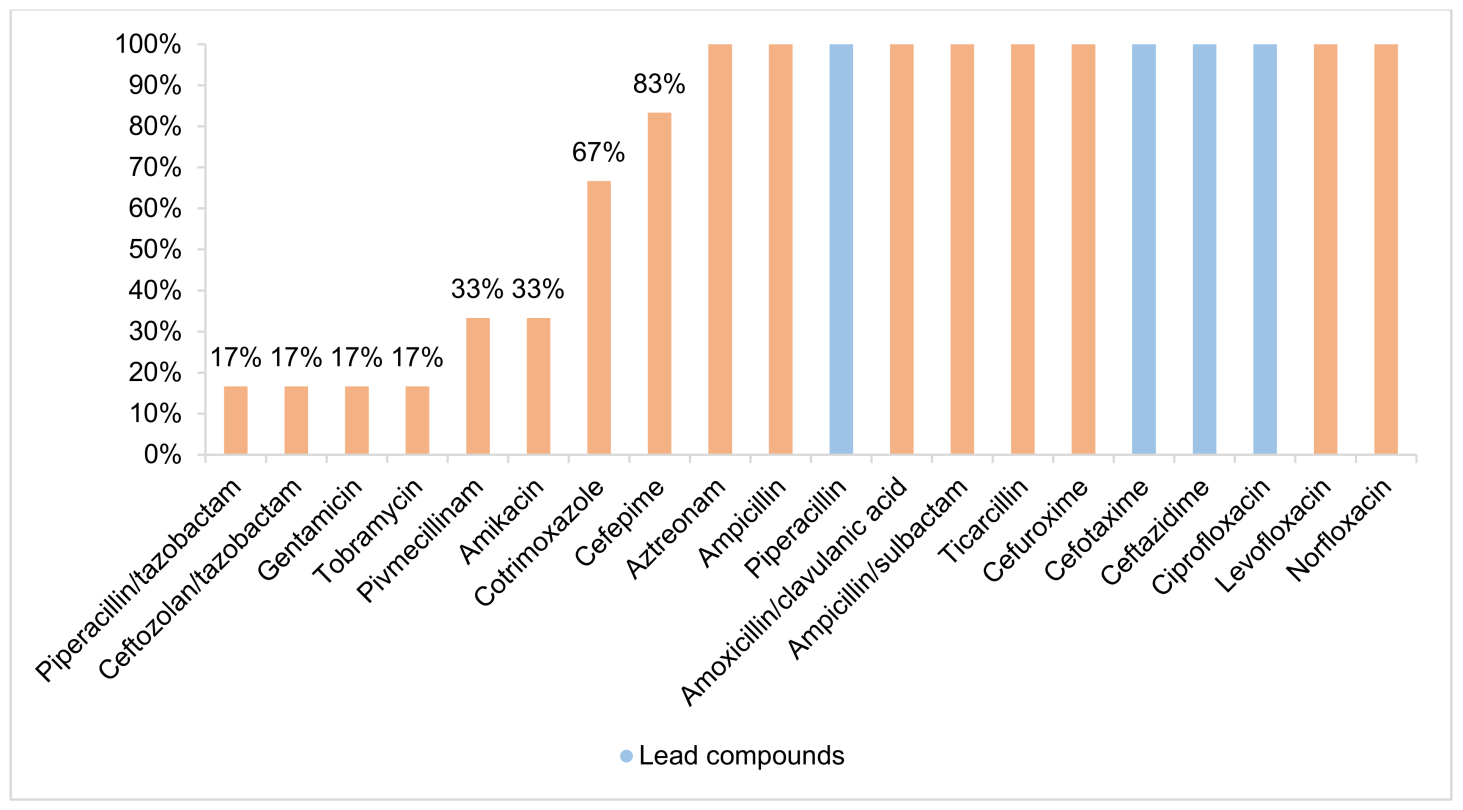
Antibiotic resistance of MRGN (evaluation according to EUCAST)

**Figure 4:**
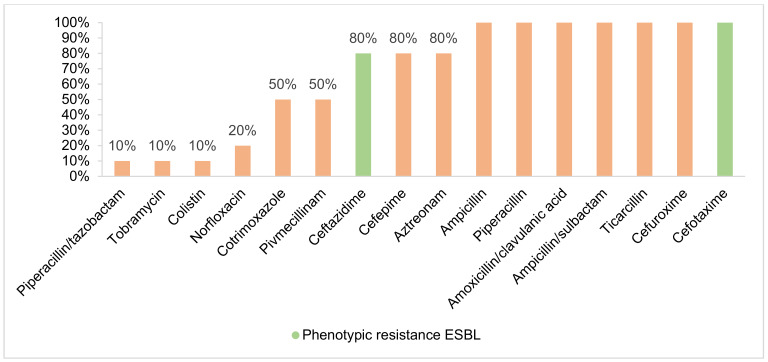
Antibiotic resistance of ESBL (evaluation according to EUCAST)

A 3MRGN prevalence of 2.60% (6/231; 95% CI 1.06–5.68) was found in the absence of 4MRGN detection (Figure 3 (Fig. 3)). Of all 3MRGN, a total of five *Escherichia coli (E. coli)* and one *Klebsiella pneumoniae* were detected. The sole significant risk factor for MRGN acquisition was antibiotic use during the previous six months (odds ratio (OR) 31.83; 95% CI 3.59–281.96; p-value 0.002). A total of 5.63% of the strains (Figure 4 (Fig. 4)), all of which were *E. coli*, formed ESBL (13/231; 95% CI 3.23–9.47). A significant risk factor for acquiring ESBL prior to travel was “male gender” (odds ratio: 4.32; 95% CI 1.16–16.12; p-value 0.030). Four of the ESBL-producing *E. coli* could also be classified as 3MRGN. The median age of the MRGN group was approximately eight years higher than in the ESBL group, without statistical significance (Table 1 (Tab. 1)). With regard to hygiene behaviour, five participants (83.33%, p-value 0.952) in the MRGN-positive group took disinfectants with them on their trip, as did nine participants (69.23%, p-value 0.139) in the ESBL-positive group (Table 1 (Tab. 1)). 

A search using the search parameter “ESBL Germany” in PubMed yielded a total of 285 studies. After focusing on “EBSL Germany travel” in a study of travellers, 19 studies appeared, which were supplemented with the results from Google Scholar (n=47, Figure 1 (Fig. 1)) (as of 24 October 2024). A total of 91 studies were found when searching for MRGN prevalence (Figure 1 (Fig. 1)). After applying the exclusion criteria, five ESBL studies and five MRGN studies were ultimately included (Figure 1 (Fig. 1)).

In general, there were many studies from the Rhine-Main region [20], [21], [22], [23]. 

The six included studies from the field of rehabilitation and in hospitals, nursing and care homes and medical practices had varying numbers of participants for anal-/rectal swab or stool analysis (range: 156–1,549, Table 2 (Tab. 2)). Four included studies with selective data from the outpatient setting had lower case numbers (range: 132–527, Table 2 (Tab. 2)). 

A literature review on the resistance situation in different populations and risk settings in various federal states revealed high 3MRGN prevalences, particularly in nursing and care homes, with 7.2% [24] in Saxony and 12.3% [20] in the Rhine-Main area (Figure 2 (Fig. 2)). Comparatively high 3MRGN prevalences were also found in hospitals and rehabilitation centres, with 7.1% [21] in the Rhine-Main area and 5.6% [24] in Saxony. In the outpatient population and among travellers, only a few studies on MRGN prevalence were included, with prevalence rates of 3.2% 3MRGN in medical practices in Saxony [24] and 7.6% 3MRGN in outpatient care services [22] in Hesse. ESBL prevalence in the outpatient setting was 5.5% ESBL Enterobacterales [25] in the outpatient population in Lower Saxony, 3.0% among travellers [26] in Saxony, 6.8% among outpatient care services in the Rhine-Main area [22] and 8.3% among travellers in Westphalia [27] (Figure 1 (Fig. 1), Table 2 (Tab. 2)).

## Discussion

The establishment of national hygiene rules is dependent on regional data on the prevalence of resistance being available nationwide for decision-making purposes. In this study and literature review, resistance data was collected in Saarland and classified according to currently known national data. This revealed a lack of comprehensive geographical MRGN data, particularly in the outpatient sector. MRGN analysis is important for deriving hygiene measures and implementing preventive measures, e.g. final disinfection of the examination room [28]. 

The MRGN definition established in Germany and Austria is often not used due to its limited international comparability. As a result, only 19% of the data from Germany was evaluated for MRGN (Figure 1 (Fig. 1)). This reduces the supraregional usability of the data collection and international comparison. At the same time, the national significance of the ESBL data is limited, as the KRINKO recommendations are based in particular on the MRGN classification [9].

The 3MRGN prevalence of 2.6% from Saarland was similar to that reported by Sommer et al. [24], which was 3.2% in a data collection from medical practices in Saxony. A higher 3MRGN prevalence of 7.6% was found in outpatient care services in Hesse [22]. The ESBL prevalence in Saarland of 5.6%, which was determined in the context of this study, was comparable to that of Symanzik et al. [25] with 5.5% in the outpatient population in Lower Saxony. 

The literature review showed that MRGN prevalence in Germany was often surveyed with a focus on risk populations and structures, with higher prevalence rates in elderly care in particular, with up to 17.8% ESBL and 12.3% 3MRGN [20]. This could be due to the fact that there are more known MRGN risk factors in this sector, such as more frequent hospital contacts. 

Multicentre studies often could not be assigned to specific federal states, making it impossible to assign geographical prevalence [29] and complicating the derivation of regionally adapted hygiene measures. Furthermore, it became apparent that some studies focused on selected resistance patterns, such as Rhode et al. [29] on third-generation cephalosporin-resistant Enterobacterales. Furthermore, there were many multicentre studies that mapped tertiary care with a specific risk profile, as these settings are characterised by more frequent high-risk procedures with lengthy hospital stays [9]. In addition, there were specific study groups, such as refugees or agricultural workers, for whom no data transfer to general prevalences could be made due to their region of origin or the divergent use of antibiotics in agriculture [6]. Individual studies also referred to a larger geographical area and not a specific federal state, such as Rodríguez-Molina et al. [30] on the greater southern Germany area. One factor that made it difficult to compare the studies was the test material, because stool samples or anal/rectal swabs were not always used; instead, data from the Hospital Infection Surveillance System (KISS) was used.

There are striking geographical differences depending on the area and federal state, and in approximately half of the federal states (including the Rhine-Main area with Bavaria, Hesse and Rhineland-Palatinate), the relevant information is not available due to a lack of surveys or data publication (Figure 2 (Fig. 2)). Comprehensive data from other federal states in all of the above-mentioned areas could not be obtained from the literature. In Saarland in particular, there are few submissions from inpatient and outpatient care to the RKI’s Antibiotic Resistance Surveillance (ARS) [31], which is why the present study from a travel medicine outpatient clinic with a 3MRGN rate of 2.6% and an ESBL prevalence of 5.6% provides new data. The comparison of data from the RKI’s Antibiotic Resistance Surveillance (ARS) on MRGN in various areas is limited, as ARS data has not been evaluated in this regard since 2014 because the MRGN definition was introduced primarily to define hygiene measures [9] and not for epidemiological surveillance. The calculation of MRGN rates was based on the antibiograms of the isolates. However, as values for the defining antibiotic groups were increasingly missing, these antibiograms were increasingly unsuitable for reliable analysis (information from personal communication with the RKI). Overall, this results in a lack of data. 

The lower number of cases in outpatient studies could be due, among other things, to the difficulty of recruiting study participants compared to the inpatient sector and rehabilitation. Furthermore, it must be discussed whether there was a need for data, as MRGN prevention measures and diagnostics have so far been used primarily in inpatient settings and rehabilitation. This is because contact isolation and other hygiene measures are indicated in inpatient settings depending on the area, pathogen and high-risk patients [9]. As a result of rising life expectancy, with an increase in outpatient care services and a decrease in the length of inpatient stays [32], it is all the more important to focus on the transmission of resistance in the outpatient sector and to evaluate the existing risk and derive hygiene measures. Outpatient colonisation poses a risk of resistance transmission to the inpatient sector and further spread there [10]. Nevertheless, there has been a low number of outpatient screenings in German emergency departments to date [33]. The lower MRGN admission prevalence in the case of risk-based screening compared to an analysis of all patients admitted to intensive care units suggests that there are MRGN-positive patients outside the risk groups and indicates that the defined risk factors are not specific enough [34].

In outpatient settings, there are currently no specific recommendations for colonised individuals, as hygiene measures such as isolation are often difficult to implement due to structural conditions. In future, when designing or redesigning outpatient facilities, the necessary spatial provisions for colonised patients should be taken into account (e.g. extra waiting areas, waiting times due to disinfection measures) and included in corresponding recommendations for the outpatient sector. Alternative prevention concepts that reduce resistance overall, such as national campaigns to promote hand hygiene [35], are also particularly important in the outpatient sector. 

Close physical contact, including in the household [36], [37], is an important risk factor for the transmission of resistance [4] and should be considered as an outpatient hygiene measure in the population for successful implementation, in particular through regular training for high-risk patients, their relatives and staff. One simple alternative would be to introduce a buddy principle, whereby two people support each other in complying with hygiene measures, observe each other and correct each other if necessary. 

It would be important to investigate such concepts prospectively in order to establish hygiene measures for risk groups on a permanent basis, especially in an environment that could become more difficult for patients from an organisational point of view with increasing outpatient health services. 

Increasing MRGN prevalence [38] is caused by many factors, including travel [14], agricultural [6] and antibiotic use in human medicine [4]. In the first two years of the COVID-19 pandemic, a significant decline in the overall European consumption of systemically effective antibiotics was observed, particularly in the outpatient sector [39], with an increased use of broad-spectrum and reserve antibiotics [40]. The increased implementation of hygiene measures during the COVID-19 pandemic may be a factor influencing the transmission of resistance [41] and may occur through direct or indirect contact [9]. Infection prevention, particularly through hand hygiene, is therefore of high importance and has clearly demonstrated during the COVID-19 pandemic that it is feasible on a population-wide basis [42]. Based on this, it was discussed that the hygiene requirements of the COVID-19 pandemic, including the use of alcohol-based hand sanitisers, could lead to lower infection rates from antibiotic-resistant bacteria [43]. 

In summary, it can be said that there are no comprehensive comparative studies on MRGN prevalence in the outpatient population without known risk factors. Particularly in the outpatient sector, developments cannot be reliably tracked due to limited surveillance. The available data provide interesting insights with a 3MRGN rate of 2.6% and an ESBL prevalence of 5.6%. Overall, our cohort showed a low MRGN prevalence, which could be influenced by several factors, including data collection during the COVID-19 pandemic. As a result of rising life expectancy and an increase in outpatient services in the care and health sector [32], where an increase in MRGN prevalence is to be expected, it is important to focus on detecting outpatient resistance transmission and unrecognised colonisation factors. However, due to its lower international relevance compared to ESBL, the data collected is analysed less in terms of MRGN prevalence. Following the lifting of travel restrictions imposed during the COVID-19 pandemic, it can be assumed that travellers could once again play a greater role in outpatient transmission.

Risk factors such as carrying disinfectants (Table 1 (Tab. 1)) probably reflect different risk behaviour in relation to the COVID-19 pandemic than before the pandemic. Carrying disinfectants suggests an increased awareness of hygiene. On the one hand, this limits the comparability of the data presented in the literature review, but on the other hand, the data are particularly interesting with regard to this influencing factor, as they show that patients implement appropriate behaviour when given appropriate training and provided with hygiene materials. 

The comparability of data from the literature review is limited by the fact that study criteria are not always identical, particularly due to differences in study timing, settings and methods (especially different resistance tests), so that although nursing homes could be classified as outpatient settings, they were considered separately due to MRGN risk factors in order to minimise potential consumption. This highlights the importance of clear guidelines for MRGN testing in the geographical setting. 

The exclusion rate of the screening performed may have been influenced by the effort involved in collecting stool samples. In addition, study inclusion was made more difficult by COVID-19 travel restrictions. Study recruitment in the travel clinic may reflect specific risk behaviour for MRGN acquisition, although this should be considered with the limitation that all travellers required a SARS-CoV-2 PCR test. This also resulted in travellers presenting who would not normally visit a travel medicine clinic and who could therefore be assumed to have lower prevention behaviour. The risk factors analysed in this study may be distorted by the low MRGN and ESBL prevalences and were somewhat lower compared to similar study settings (Figure 2 (Fig. 2)), which may have been influenced by COVID-19 measures. 

## Conclusion

Due to its international relevance, ESBL data is often collected and published in Germany in an outpatient setting instead of MRGN data. There is only limited epidemiological data available on MRGN prevalence in all healthcare structures and federal states, which is why this study, with an outpatient study population in Saarland, represents previously insufficiently known resistance data in a regional comparison and shows that hygiene implications could arise due to internationally varying definitions of resistance. The increasing structural shift towards outpatient care in the healthcare system and the growing relevance of MRGN highlight the need for rigorous analysis and implementation of preventive measures with detailed, comprehensive surveillance in terms of geography, risk factor detection and different care sectors. 

## Notes

### Competing interests

C. Berdin, S. Schneitler, T. Kaspers, B. Gärtner, A. Halfmann, S. L. Becker, F. Berger and N. Walzer: in general no conflict of interest. Just as a general information, a small portion of the underlying data were previously included in Kaspers et al. [44] as part of the overall project, though under a different evaluative framework and to address separate research questions. Parts of the study were funded by start-up financing from the state of Saarland. S. Schneitler is involved in voluntary work for the German Society for Tropical Medicine, Travel Medicine and Global Health (Deutsche Gesellschaft für Tropenmedizin, Reisemedizin und Globale Gesundheit e.V.) and the Network for Young Infectious Medicine (Netzwerk junge Infektionsmedizin e.V.).

## Figures and Tables

**Table 1:**
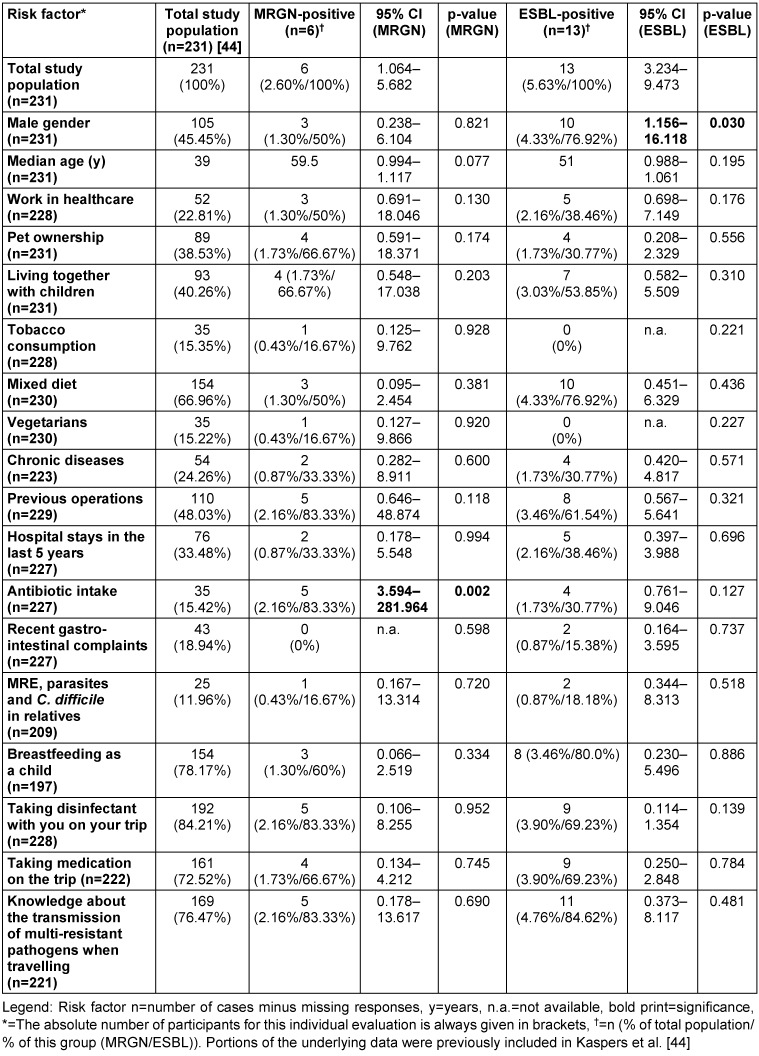
Comparison of individual risk factors analysed for all 3MRGN (five *E. coli* + 1 *Klebsiella pneumoniae*) and all ESBL *E. coli* with illustration of the total cohort

**Table 2:**
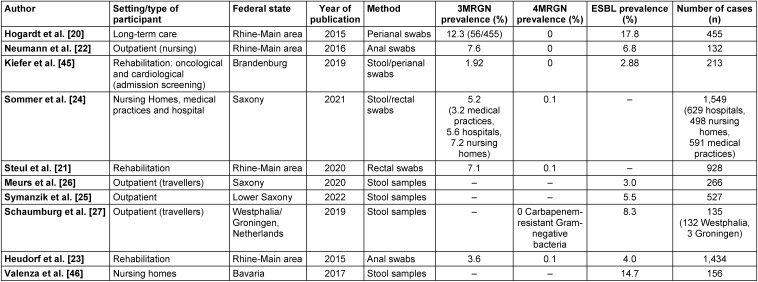
Results of the literature review (areas, federal states, prevalence and case numbers). n refers to collected stool samples and perianal swabs. In some studies, n may also include results obtained from other swab types.
